# Stable isotope dilution assay for the accurate determination of mycotoxins in maize by UHPLC-MS/MS

**DOI:** 10.1007/s00216-012-5757-5

**Published:** 2012-02-02

**Authors:** Elisabeth Varga, Thomas Glauner, Robert Köppen, Katharina Mayer, Michael Sulyok, Rainer Schuhmacher, Rudolf Krska, Franz Berthiller

**Affiliations:** 1 Christian Doppler Laboratory for Mycotoxin Metabolism and Center for Analytical Chemistry, Department for Agrobiotechnology (IFA-Tulln), University of Natural Resources and Life Sciences Vienna, Konrad Lorenz Str. 20, 3430 Tulln, Austria; 2 Chemical Analysis Group, Agilent Technologies Sales & Services GmbH & Co. KG, Hewlett-Packard-Str. 8, 76337 Waldbronn, Germany; 3 Division 1.2 Organic Chemical Analysis, Reference Materials, Federal Institute for Materials Research and Testing, Richard-Willstätter-Str. 11, 12489 Berlin, Germany

**Keywords:** Mycotoxins, Stable isotope dilution assay, Multi-target analysis, Ultrahigh-performance liquid chromatography, Tandem mass spectrometry, Maize

## Abstract

A fast, easy-to-handle and cost-effective analytical method for 11 mycotoxins currently regulated in maize and other cereal-based food products in Europe was developed and validated for maize. The method is based on two extraction steps using different acidified acetonitrile–water mixtures. Separation is achieved using ultrahigh-performance liquid chromatography (UHPLC) by a linear water–methanol gradient. After electrospray ionisation, tandem mass spectrometric detection is performed in dynamic multiple reaction monitoring mode. Since accurate mass spectrometric quantification is hampered by matrix effects, uniformly [^13^C]-labelled mycotoxins for each of the 11 compounds were added to the sample extracts prior to UHPLC-MS/MS analysis. Method performance parameters were obtained by spiking blank maize samples with mycotoxins before as well as after extraction on six levels in triplicates. The twofold extraction led to total recoveries of the extraction steps between 97% and 111% for all target analytes, including fumonisins. The [^13^C]-labelled internal standards efficiently compensated all matrix effects in electrospray ionisation, leading to apparent recoveries between 88% and 105% with reasonable additional costs. The relative standard deviations of the whole method were between 4% and 11% for all analytes. The trueness of the method was verified by the measurement of several maize test materials with well-characterized concentrations. In conclusion, the developed method is capable of determining all regulated mycotoxins in maize and presuming similar matrix effects and extraction recovery also in other cereal-based foods.

## Introduction

Mycotoxins are low-molecular-weight secondary metabolites of fungi which can cause immunosuppressive, hepatotoxic, mutagenic, carcinogenic, or estrogenic effects in mammals [1]. Nowadays, hundreds of mycotoxins have been identified, but only about a dozen are considered to be of major concern regarding their occurrence and toxicity. According to van Egmond et al. [2], in 2003, at least 99 countries had regulations for mycotoxins in food and/or feed. The European Union Commission Regulation 1881/2006 and its amendments [3–6] set maximum levels (MLs) for 11 mycotoxins in food: aflatoxins (the sum of aflatoxin B_1_, B_2_, G_1_ and G_2_ (AFB_1_, AFB_2_, AFG_1_, AFG_2_) as well as AFB_1_ alone and aflatoxin M_1_); the sum of fumonisin B_1_ and B_2_ (FB_1_, FB_2_); ochratoxin A (OTA); patulin; deoxynivalenol (DON); and zearalenone (ZEN). The MLs for HT-2 and T-2 toxin (HT-2 and T-2) are under preparation and will be available shortly.

To ensure the enforcement of these limits, reliable and accurate analytical methods are necessary. So far, most of the official methods for the determination of mycotoxins in food or feed are single-target analyte methods, and none of them is based on LC-MS/MS. However, tandem mass spectrometry is a powerful tool for the analysis of mycotoxins with high sensitivity and selectivity. A comprehensive review about the analysis of mycotoxins at trace levels by LC-MS/MS is provided by [7], and an overview of the multi-target methods for the simultaneous determination of mycotoxins in different commodities in recent years is given by [8].

Matrix effects are caused by the suppression or enhancement of the analyte signal during the ionisation process and may hamper the accurate quantification, leading to incorrect results. A review dealing with matrix effects in LC-MS/MS methods was recently published [9]. Prediction of matrix effects is difficult because they are influenced by several factors like target compound (chemical structure, polarity), matrix type and the relative concentrations of the substances competing for the limited number of charges. Additionally, sample preparation (extraction process, cleanup), chromatographic conditions, mass spectrometric instrumentation (e.g. design of ion source) and ionisation conditions influence the extent and precision of matrix effects [9]. Ion suppression may be caused by the presence of matrix compounds co-eluting with the target analytes and reducing the ion intensity as well as effecting the reproducibility and accuracy of the assay [10]. Substances which affect the boiling point or the surface tension of the LC eluent or are affecting the droplet size during ionisation have the potential to change the concentration of the target analyte in the gas phase and hence lead to matrix effects [11]. Huge differences in the degree of matrix effects are not only seen for different matrices; high variances between individual samples of one matrix type were also observed [12]. On top of that, even after extensive sample cleanup or quite specific cleanups like immunoaffinity columns, severe matrix effects were reported [13].

Possible approaches to cope with matrix effects are dilution of the sample, matrix-matched calibration, standard addition or internal calibration. Dilution of the sample obviously reduces the sensitivity of the analytical method. Whilst matrix-matched calibrations are extensively applied, they are tedious and differences within a given commodity cannot be fully compensated. Additionally, for some analytes, it might be difficult to find a blank matrix for spiking (e.g. DON in maize). Standard addition to each sample is also often used in routine analysis, but at least doubles the number of LC runs [14]. In the case of internal calibration, the ideal internal standard behaves exactly like the target analyte, but is still distinctive. In practice, structural related or similar compounds, as well as stable isotopically labelled compounds, are used as internal standards. For example, zearalanone (ZAN), differing to ZEN only in the absence of one double bond, was used to compensate the matrix effects of ZEN (e.g. [15]) or of several mycotoxins (e.g. [16]). Verrucarol (e.g. [17]) and deepoxy-deoxynivalenol (e.g. [18]) were used as internal standards for A- and B-trichothecenes. A drawback of all structurally different compounds is that they do not co-elute and may behave differently during analysis, and hence may lead to false results.

Stable isotopically labelled standards share the same chemical and physical properties as the target analytes, but are still distinct over their different molecular mass. Additionally, they are not present in naturally contaminated samples. Since the naturally abundant isotopic distribution of the analyte is diluted due to the addition of stable isotope-labelled standards, this procedure is often referred to as stable isotope dilution assay (SIDA). A review of the application of SIDA in mycotoxin analysis was published by Rychlik and Asam in 2008 [19]. In general, the use of [^13^C]- or [^15^N]-labelled compounds is preferred over deuterium [^2^H] or [^18^O] labels. Carbon and nitrogen are often part of the molecule backbone, and C–C or C–N bonds are less likely to be cleaved. In the case of deuterated compounds, H–D exchanges with the surrounding solution are frequent. Additionally, so-called isotope effects, small physical or chemical differences of isotopologues that even may lead to a slight shift in retention time in the case of deuterium-labelled compounds [20], are less prone for [^13^C] and [^15^N] than for [^2^H]. For small organic molecules, a mass increase of at least 3 between the naturally occurring compound and the stable isotope-labelled analogue is recommended [19].

Several SIDA methods have been developed to quantify single mycotoxins in food. For instance, [^2^H_6_]-FB_1_ was used for the determination of FB_1_ in maize products [21], whilst [^13^C]-labelled fumonisins were used for the analysis of traditional Chinese medicines [22]. [^13^C_2_]-labelled T-2, HT-2, diacetoxyscirpenol and monoacetoxyscirpenol were used for the quantification of type A-trichothecenes in food and feed [23]. Uniformly labelled U-[^13^C_24_] T-2 toxin was applied as the internal standard for the analysis of maize and oats [24]. 15-[^2^H_1_]-DON and 3-[^2^H_3_]-acetyldeoxynivalenol were used for the determination of DON and 3-acetyldeoxynivalenol in cereals [25]. The application of U-[^13^C_15_]-labelled DON for the determination of DON in maize and wheat without any sample cleanup was shown in 2006 [26]. The first SIDA method for multiple B-trichothecenes (DON, 3-acetyldeoxynivalenol (3-ADON), 15-acetyldeoxynivalenol (15-ADON) and fusarenon X (FusX)) was published in 2007 [27] using [^13^C_15_]-DON, [^13^C_2_]-3-ADON, [^13^C_2_]-15-ADON and [^13^C_2_]-FusX as internal standards.

[3,5-^2^H_2_]-ZEN synthesis and its subsequent use as an internal standard for ZEN determination in cereal products were presented [28]. U-[^13^C_20_]-labelled OTA was used for the determination of OTA in red paprika [29], whilst [^2^H_5_]-labelled OTA was used as the internal standard in food analysis [30]. A SIDA method for aflatoxins in different food products was developed by Cervino et al. [31] who gained [^2^H_2_]-AFB_2_ and [^2^H_2-4_]-AFG_2_ by a catalytic deuteration of AFB_1_ and AFG_1_. For the determination of the four major aflatoxins in animal feed by solid phase extraction, U-[^13^C_17_]-AFB_1_ was used as the internal standard for all analytes [32].

Matrix effects and several compensation strategies including stable isotope dilution assays were evaluated [33]. As internal standards, [^2^H_1_]-DON, U-[^13^C_24_]-T-2, U-[^13^C_22_]-HT-2, U-[^13^C_34_]-FB_1_ and U-[^13^C_34_]-FB_2_ were applied in two multi-methods (one for fumonisins and one for trichothecenes and ZEN), and ZAN was used as a cleanup standard compensating the losses of ZEN during sample preparation. For routine analysis, the use of standard addition or internal standards is preferred to matrix-matched calibration since differences within one matrix are not compensated by the latter.

Until recently, internal standards were only used in single-target or group target methods or in multi-methods using a limited number of internal standards. However, the results from one internal standard–mycotoxin combination are not suitable to another mycotoxin of a different chemical property and/or different retention time. Hence, an own internal standard for each analyte is required. Zachariasova et al. [34] developed a multi-method using high-resolution mass spectrometry for the analysis of cereals for 11 major *Fusarium* mycotoxins (DON, HT-2, T-2, ZEN, FB_1_, FB_2_, fumonisin B_3_ (FB_3_), nivalenol, deoxynivalenol-3-glucoside (D3G), 3-ADON, FusX). Eight commercially available [^13^C]-labelled standards (excluding D3G and FusX) were applied to the sample before extraction to compensate losses during cleanup and ionisation [34]. Lattanzio et al. [35] recently published a SIDA method using U-[^13^C]-labelled standards for the determination of nine mycotoxins (AFB_1_, AFB_2_, AFG_1_, AFG_2_, OTA, DON, ZEN, T-2 and HT-2) in cereal-based foods. After extraction with an acetonitrile–water mixture, a cleanup step with a polymeric solid phase extraction followed and the internal standards were applied before LC-MS/MS analysis [35].

The aim of this work was to establish a fast, reliable and easy-to-handle ultrahigh-performance liquid chromatography (UHPLC)-MS/MS method for the accurate determination of mycotoxins regulated in the European Union in maize and cereal-based foodstuff. The method includes the determination of aflatoxins (AFB_1_, AFB_2_, AFG_1_, AFG_2_), fumonisins (FB_1_, FB_2_), OTA, DON and ZEN, as well as T-2 and HT-2. The accuracy was enhanced by the application of U-[^13^C]-labelled compounds for each of the target analyte prior to UHPLC-MS/MS analysis. Method performance parameters have been evaluated for maize. This is the first method for the determination of all aforementioned mycotoxins in maize using stable isotope dilution mass spectrometry.

## Experimental

### Reagents and maize samples

Methanol and acetonitrile (Baker analysed LC-MS reagent) were purchased from JT Baker (Deventer, the Netherlands). Formic acid and ammonium formate (both LC-MS grade) were purchased from Sigma Aldrich (Vienna, Austria). Water was purified by reverse osmosis and a subsequent Milli-Q-plus system from Millipore (Molsheim, France).

All standards including the unlabelled and the U-[^13^C]-labelled mycotoxins were purchased from Romer Labs GmbH (Tulln, Austria). Besides the unlabelled aflatoxins and fumonisins, which were two combined solutions, all standards were individual stock solutions in acetonitrile or acetonitrile/water (1:1, *v*/*v*). Both the stocks of the unlabelled and the U-[^13^C]-labelled compounds were combined to two working solutions (one for the unlabelled mycotoxins in acetonitrile and one for the [^13^C]-labelled analogues in acetonitrile/water (28:72, *v*/*v*)). The working solution of the unlabelled mycotoxins had the following concentrations: AFB_1_, AFB_2_, AFG_1_ and AFG_2_, 750 ng mL^−1^; DON, 7,510 ng mL^−1^; FB_1_, 2,490 ng mL^−1^; FB_2_, 2,510 ng mL^−1^; HT-2, 6,720 ng mL^−1^; OTA, 765 ng mL^−1^; T-2, 754 ng mL^−1^; ZEN, 2,520 ng mL^−1^. The concentrations of the [^13^C]-labelled working solutions were as follows: [^13^C_17_]-AFB_1_, 10.6 ng mL^−1^; [^13^C_17_]-AFG_1_, 10.4 ng mL^−1^; [^13^C_17_]-AFB_2_ and [^13^C_17_]-AFG_2_, 10.0 ng mL^−1^; [^13^C_15_]-DON, 500 ng mL^−1^; [^13^C_34_]-FB_1_, 502 ng mL^−1^; [^13^C_34_]-FB_2_ and [^13^C_22_]-HT-2, 508 ng mL^−1^; [^13^C_20_]-OTA, [^13^C_24_]-T-2 and [^13^C_18_]-ZEN, 50.0 ng mL^−1^. The individual stock solutions and the two working solutions were stored at −20 °C. Prior to usage, the working solutions were brought to room temperature in the dark and were mixed thoroughly. Neat standard solutions were obtained by the dilution of the unlabelled working solution with a dilution solvent (acetonitrile/water, 30:70, *v*/*v*). For the preparation of the standards, 80 μL of the neat standard solutions were transferred into HPLC vials with microinserts (VWR International, Leuven, Belgium) and 20 μL of the [^13^C]-labelled standard working solution was added. This resulted in the following concentrations for the internal standards in the vials: [^13^C_17_]-AFB_1_ and [^13^C_17_]-AFG_1_, 2.1 ng mL^−1^; [^13^C_17_]-AFB_2_ and [^13^C_17_]-AFG_2_, 2.0 ng mL^−1^; [^13^C_15_]-DON and [^13^C_34_]-FB_1_, 100 ng mL^−1^; [^13^C_34_]-FB_2_ and [^13^C_22_]-HT-2, 102 ng mL^−1^; [^13^C_20_]-OTA, [^13^C_24_]-T-2 and [^13^C_18_]-ZEN, 10.0 ng mL^−1^. Standards were measured in increasing concentrations with one blank before and two blanks afterwards.

Maize for spiking experiments was visually inspected for the absence of mould, ground and thoroughly mixed. After extraction, the maize was measured using LC-MS/MS proving contamination with mycotoxins below the limit of detection (LOD). Twelve maize test materials (TM) including reference materials (RM), check sample materials (CSM) and samples from diverse ring trials were purchased (internal numbering in parentheses): EU Joint Research Centre (JRC) BCR maize no. 717 (TM_01), Food Analysis Performance Assessment Scheme (FAPAS) maize T04138 (TM_02), FAPAS maize T2246 (TM_03), FAPAS maize T2262 (TM_04), Romer Labs—CSM maize no. BRM 003024 (TM_05), Romer Labs—CSM maize no. BRM 003017 (TM_06), Romer Labs—CSM maize no. BRM 003018 (TM_07), Romer Labs—CSM maize no. BRM 003010 (TM_08), Romer Labs—RM maize no. BRM 003011 (TM_09), Scientific and Technological Research Council of Turkey—Ankara Test and Analysis Laboratory (TÜBITAK-ATAL)/JRC maize level 1 (TM_10), TÜBITAK-ATAL/JRC maize level 2 (TM_11), TÜBITAK-ATAL/JRC maize level 3 (TM_12).

### Sample preparation

Ground and homogenized maize samples (5.00 ± 0.01 g) were weighed into 50-mL polypropylene tubes (VWR International). The first extraction was performed with the fourfold amount (20 mL) of extraction solvent 1 (acetonitrile/water/formic acid, 80:19.9:0.1, *v*/*v*/*v*) on an Edmund Bühler GmbH SM30 rotary shaker (Hechingen, Germany) for 60 min at room temperature. After extraction, the tubes were centrifuged for 5 min (3,500 rpm) with an Eppendorf AG Centrifuge 5804 R (Hamburg, Germany) and the raw extract decanted into a new 50-mL polypropylene tube (VWR International). The residue was extracted a second time with 20 mL extraction solvent 2 (acetonitrile/water/formic acid, 20:79.9:0.1, *v*/*v*/*v*) on the rotary shaker for 30 min at room temperature. Afterwards, the samples were centrifuged again for 5 min (3,500 rpm) and the supernatant combined with the first extract. The solid residue of the second extraction step was discarded and the combined extract was centrifuged again for 5 min (3,500 rpm). An aliquot (80 μL) of the centrifuged raw extract was transferred into an HPLC vial with a microinsert (VWR International) and 20 μL of the [^13^C]-labelled working solution added. The content of the vial was mixed and 3 μL thereof was injected directly into the UHPLC-MS/MS system. This way, for instance, 10 ng mL^−1^ corresponds to 100 μg kg^−1^ (conversion factor of 10).

### UHPLC-MS/MS parameters

For analysis, a 1290 series UHPLC system coupled to a 6490 Triple Quadrupole (QqQ) mass spectrometer (both Agilent Technologies, Waldbronn, Germany) was used. The 6490 QqQ system was equipped with an Agilent Jet-Stream ESI interface and was operated by MassHunter Workstation B.04.01 software. Precursor and product ion selection as well as the optimization of collision energies were performed with flow injection of single analyte solutions using the MassHunter Optimizer software. The used analytical column was a ZORBAX RRHD Eclipse Plus C18 (100 × 2.1 mm, 1.8 μm) column from Agilent Technologies. Chromatographic separation was performed at 30 °C with a flow rate of 350 μL min^−1^. Eluent A was composed of water/formic acid (99.9:0.1, *v*/*v*) and eluent B of methanol/formic acid (99.9:0.1, *v*/*v*); both contained 5 mM ammonium formate. The total run time of the chromatographic run was 11.5 min comprising an initial hold time of 0.5 min at 30% B and a linear gradient to 100% B within 7.5 min. After a hold time of 1.5 min at 100% B, the starting composition of 30% B was reached within 0.1 min and hold for 2 min to allow column re-equilibration. The eluent flow within the first minute of injection and from 7 min until the end of the analysis was directed to the waste via the column compartment selection valve. Before sample injection, the needle was washed in the flush port with acetonitrile/water (50:50, *v*/*v*) for 5 s.

Analysis was carried out using the dynamic multiple reaction monitoring mode and fast polarity switching. Monitoring of two multiple reaction monitoring (MRM) transitions (quantifier and qualifier) resulted in 4.0 identification points (IPs). This is in agreement with Commission Decision 2002/657/EC [36] in which 3.0 IPs are required for the identification of mycotoxins in food. The general source settings in the positive (pos.) and negative (neg.) ionisation modes were as follows: gas temperature, 140 °C; gas flow, 16 L min^−1^; nebulizer, 25 psi; sheath gas temperature, 350 °C; sheath gas flow, 11 L min^−1^; capillary voltage, 4,000 V (pos.) and 3,000 V (neg.); and nozzle voltage, 0 V. The fragmentor voltage was 380 V for all mass transitions, and both scanning quadrupoles (Q1 and Q3) were set to unit resolution.

### Method validation

The determined method performance parameters include apparent recovery, matrix effects and extraction recovery. Additionally, the working range, limits of detection and quantification, as well as repeatability and trueness of the method have been evaluated for maize. For the determination of all parameters, the guidelines of Commission Decision 2002/657/EC [36] were taken into account.

Recovery experiments were performed by spiking blank maize samples (5.00 ± 0.01 g) with the appropriate amount of spiking solution (unlabelled mycotoxins) on six levels in triplicate before extraction. For the three highest levels, the working solution of the unlabelled mycotoxins was used as the spiking solution (1,000, 300 and 100 μL, respectively). For the lower levels, the working solution was diluted 1:10 with acetonitrile, and 300, 100 and 30 μL were used for the spiking experiment. Spiked samples were stored uncapped overnight at room temperature to allow solvent evaporation and to achieve equilibrium between the analytes and matrix. On the next day, the samples were capped followed by a short shaking by hand to ensure a homogenous distribution of the spiked maize. For further sample preparation, the above protocol was followed.

To evaluate matrix effects, blank maize samples were extracted and matrix-matched standards were prepared on six levels in triplicates. An aliquot of the raw extract (975 μL) was combined with 25 μL working solution of the unlabelled mycotoxins and was thoroughly mixed. Further dilutions of this spiked raw extract were performed with blank raw extract to obtain in total six spiking levels with relative concentrations of 1:3.33:10:33.3:100:333 (see Table 2 for the concentration range of the single analytes). Of the spiked raw extracts, 80 μL was combined with 20 μL of the U-[^13^C]-labelled working solutions, and 3 μL thereof was injected directly into the UHPLC-MS/MS system. The trueness of the method was verified by the measurement of 12 test materials with well-defined analyte concentrations of different providers.

### Data evaluation

For data evaluation, 1/*x* weighted calibration curves were obtained for each analyte by plotting the relative response versus the analyte concentration using MassHunter Quantitative Analysis version B.04.00. The relative response was the peak area of the analyte signal divided by the peak area of the corresponding internal standard. For each spiking level, the observed concentrations were calculated by the relative response and the calibration curves using internal calibration. Apparent recoveries were gained by the ratio of measured to spiked concentration in per cent followed by calculating the average value of all six spiking levels and triplicate analysis.

For the evaluation of matrix effects, two procedures were applied: First, the data were analysed without considering the internal standards; hence, linear, 1/*x* weighted calibration curves were obtained by plotting peak areas versus the analyte concentrations. This approach led to the determination of the apparent recoveries with external calibration. Furthermore, signal suppression or enhancement (SSE int.) of the SIDA method was calculated from the spiked blank extracts in the same way as the apparent recovery was determined. For the calculation of the extraction recovery (*R*_E_), the obtained mean values for the apparent recovery using internal calibration (*R*_A_ int.) were divided by the obtained mean values for the signal suppression or enhancement (SSE int.). Repeatability (RSD_r_) was calculated from the triplicate analysis on the six spiking levels.

To confirm the presence of a mycotoxin in the sample, the ion ratio of the quantifier to the qualifier has to be within the set target range obtained by the standard. For analytes with a relative intensity (per cent of base peak) of above 50%, this value is ±20%; for analytes with relative intensities between 20% and 50%, ±25% is allowed, in accordance with Commission Decision 2002/657/EC [36]. Additionally, the retention time has to be within ±2.5% compared with an authentic liquid standard, and both the qualifier and quantifier transition have to be above the limit of quantification (LOQ) to allow quantification. LOD and LOQ were estimated using the signal-to-noise (S/N) ratios observed in maize sample extracts of the less intensive mass transition using MassHunter Qualitative Analysis version B.04.00. In general, the noise was determined from the baseline in a time interval of 0.2 min before the respective analyte peak in the maize sample spiked before extraction. From the spiking level closest to S/N ratios of 3 and 10, respectively, the LOD and the LOQ were calculated.

## Results and discussion

### Development of the analytical method

Mass spectrometric parameters including the determination of precursor and product ions along with the corresponding optimized collision energies and cell acceleration voltages were obtained during flow injection of single analyte solutions for the natural compounds as well as for the [^13^C] analogues. The precursor ions showing the highest abundance were in most cases protonated [M+H]^+^ species. Two of the investigated analytes (HT-2 and T-2) form adducts with ammonium ions, and ZEN was the only analyte for which the highest intensity was observed for the deprotonated [M-H]^−^ species. To assure the best possible sensitivity for all target analytes in a single chromatographic run, fast polarity switching was applied. As ZEN (neg. mode) is partly co-eluting with both OTA and FB_2_ (pos. mode), this feature was very helpful. In agreement with Commission Decision 2002/657/EC [36], for each analyte, the two mass transitions with the highest abundance were selected; one served as the qualifier and one as the quantifier ion in the further analysis. Concerning the [^13^C]-labelled internal standards, only one transition was chosen. Table 1 summarizes the optimized ESI parameters for the 11 chosen mycotoxins and their internal standards.

**Table 1:** List of analytes together with optimized ESI-MS and ESI-MS/MS parameters

Analyte	RT (min)	*m/z* precursor ion	Cell acc. (V)	*m/z* product ions (CE in V)^a^	Relative response ratio^b^
Aflatoxin B_1_	4.42	313.1 [M+H]^+^	3	241.0 (41), 285.0 (21)	84
[^13^C_17_]-aflatoxin B_1_	4.42	330.1 [M+H]^+^	3	301.1 (21)	
Aflatoxin B_2_	4.17	315.1 [M+H]^+^	3	258.9 (29), 287.0 (21)	75
[^13^C_17_]-aflatoxin B_2_	4.17	332.2 [M+H]^+^	3	303.0 (21)	
Aflatoxin G_1_	3.87	329.1 [M+H]^+^	3	243.0 (25), 200.1 (41)	67
[^13^C_17_]-aflatoxin G_1_	3.87	346.1 [M+H]^+^	5	212.2 (41)	
Aflatoxin G_2_	3.59	331.1 [M+H]^+^	3	313.0 (21), 245.1 (25)	65
[^13^C_17_]-aflatoxin G_2_	3.59	348.1 [M+H]^+^	5	259.1 (25)	
Deoxynivalenol	1.45	297.1 [M+H]^+^	3	249.0 (4), 203.0 (12)	65
[^13^C_15_]-deoxynivalenol	1.45	312.2 [M+H]^+^	3	263.1 (4)	
Fumonisin B_1_	5.55	722.4 [M+H]^+^	3	352.4 (37), 334.4 (37)	87
[^13^C_34_]-fumonisin B_1_	5.55	756.5 [M+H]^+^	3	374.4 (37)	
Fumonisin B_2_	6.46	706.4 [M+H]^+^	3	336.4 (41), 318.3 (41)	57
[^13^C_34_]-fumonisin B_2_	6.46	740.5 [M+H]^+^	3	358.3 (41)	
HT-2 toxin	5.40	442.2 [M+NH_4_]^+^	3	263.0 (9), 215.0 (13)	84
[^13^C_22_]-HT-2 toxin	5.40	464.3 [M+NH_4_]^+^	3	278.1 (9)	
Ochratoxin A	6.43	404.1 [M+H]^+^	3	238.9 (25), 102.1 (70)	40
[^13^C_20_]-ochratoxin A	6.43	424.2 [M+H]^+^	3	250.1 (25)	
T-2 toxin	5.96	484.3 [M+NH_4_]^+^	5	215.1 (9), 305.0 (8)	81
[^13^C_24_]-T-2 toxin	5.96	508.3 [M+NH_4_]^+^	5	322.1 (8)	
Zearalenone	6.44	317.1 [M-H]^−^	7	130.9 (29), 272.9 (17)	69
[^13^C_18_]-zearalenone	6.44	335.2 [M-H]^−^	7	290.0 (17)	

*RT* retention time, *Cell acc.* cell acceleration voltage

^a^Values are given in the order quantifier ion, qualifier ion (in parentheses are the corresponding collision energy (CE) settings in volts)

^b^Defined as the peak area of qualifier in per cent of the quantifier

Chromatographic separation was performed by reversed phase chromatography using acidified water and methanol as mobile phases. For fumonisins, slightly acidic conditions are necessary with regard to stable retention patterns and ionisation efficiencies, as already pointed out in previous publications, e.g. [37]. To both eluents, ammonium formate was added to suppress the formation of sodium adducts which reduce MS sensitivity of ammonium ions [38]. Sodium adducts should not be used as precursor ions because they show insufficient fragmentation patterns since the positive charge remains on the sodium ion after collision-induced dissociation. Baseline separation was required for certain analytes due to the same or very similar mass transitions for FB_3_ and FB_2_ as well as for AFG_1_ and U-[^13^C_17_]-AFB_1_, and AFG_2_ and U-[^13^C_17_]-AFB_2_.

**Fig. 1:**
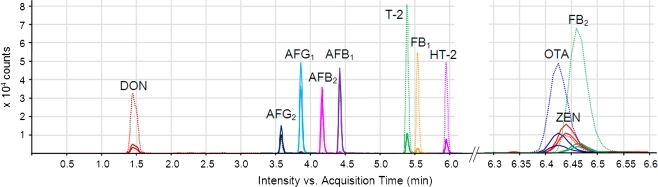
Extracted ion chromatogram of a blank maize sample spiked before extraction with unlabelled mycotoxins at a medium level (AFB_1_, AFB_2_, AFG_1_, AFG_2_, 15.0 μg kg^−1^; T-2, 15.1 μg kg^−1^; OTA, 15.3 μg kg^−1^; FB_1_, 49.8 μg kg^−1^; FB_2_, 50.1 μg kg^−1^; ZEN, 50.4 μg kg^−1^; HT-2, 134.4 μg kg^−1^; DON, 150.2 μg kg^−1^). Each analyte is displayed in a *different colour*. The *dotted lines* show the mass transitions of the internal standards, whilst the *full lines* indicate the quantifier and qualifier transitions

The use of an UHPLC instead of HPLC improves the chromatographic resolution for the target analytes and potentially reduces matrix effects by separating the target analytes and matrix. During method development, different stationary phases (Agilent ZORBAX Poroshell 120 EC-C18 and ZORBAX RRHD Eclipse Plus C18) and column lengths (50 and 100 mm), as well as column temperatures, flow rates, gradients and injection volumes, were tested with regard to resolution, peak intensity and shape as well as matrix effects (data not shown). For the final method, a ZORBAX RRHD Eclipse Plus C18 (100 × 2.1 mm, 1.8 μm) column operated at 30 °C and a flow rate of 350 μL min^−1^ were selected. The flow rate was chosen such that it is within the optimal operation values of the ESI source of the mass spectrometer and provides fast separation conditions. Figure 1 shows the overlay of the MRM transitions of a maize sample spiked at a medium level before extraction. FB_3_ is not regulated, but still occurring in naturally contaminated cereals. Due to the same mass transitions of the regulated FB_2_, it is important that these substances are not co-eluting in any given method. In our case, we easily achieved baseline separation for the two compounds with retention times of 6.06 min for FB_3_ and 6.46 min for FB_2_. The pressure varied between approximately 340 bar at 100% B and 740 bar at approximately 60% B. The total chromatographic run time was 11.5 min. The capacity factor *k*′ of the first eluting analyte (DON) is >1 (actually 1.5), a criteria set by Commission Decision 2002/657/EC [36]. It was the aim to develop an easy-to-handle method and to inject the pure extract without further dilution or change of solvent. The injection solvent after the addition of internal standard contains 46% acetonitrile. Whilst the starting conditions of the UHPLC-MS/MS method (30% methanol) possess lower elution strength than the injection solvent, injecting 3 μL led to acceptable peak shapes for all analytes.

The developed UHPLC-MS/MS method fulfils the requirements for confirmatory methods laid down in Section 2.3.3 of Commission Decision 2002/657/EC [36]. For confirmatory purposes, both mass transitions of each analyte have to be above the LOQ, the LC retention time has to be within ±2.5%, and the ion ratio within ±20% and ±25% rel., respectively, compared with the relative values of an authentic liquid standard, as already pointed out earlier. These values were set within the MassHunter Quantification software; non-compliant values are automatically flagged.

In the case of fumonisins, loss of signal intensity in neat standard solutions in microinserts occurred over time. This phenomenon was neither observed in the presence of matrix nor with standard HPLC vials. One likely explanation could be that matrix components saturate the surface of the used microinserts and that in the absence of these components, adsorption of fumonisins occurs to a small extent. Since nowadays analytical instrumentation enables the quantification of fumonisins in the low microgram per litre range, even small adsorption effects lead to an overestimation of the mycotoxin contamination due to the reduction of the standard concentration. Since the use of microinserts is feasible due to the reduced additional costs for the internal standards, four different inserts were tested. VWR International microinserts showed the least influence over time and were therefore selected (data not shown). In conclusion, special care in the choice of HPLC microinserts is necessary and long-term storage of highly diluted fumonisin solutions in microinserts is not recommended.

### Optimization of sample preparation

Extraction tests for regulated mycotoxins were already performed by various research groups [13, 38, 39], and no single extraction step capable of extracting all target analytes with high efficiency was identified. The extraction solvent extensively used for the analysis of mycotoxins consists of acidified acetonitrile/water mixtures with an acetonitrile content of about 80% (e.g. [38]). This mixture shows very good extraction recoveries for all target analytes, except fumonisins (e.g. FB_1_ of 57% and for FB_2_ of 67% in maize) [38]. The use of isotope-labelled internal standards before extraction was not considered as a viable option due to the high costs associated with the high amounts of internal standards needed. As a consequence, the extraction recovery of the developed method has to be very high. Several extraction procedures have been tested (data not shown). For instance, double and triple extractions with acetonitrile/water/formic acid (80:19.9:0.1, *v*/*v*/*v*) were performed. In addition, the described two-step extraction was used and has been superior to the above because of less total volume (resulting in less dilution) and quicker handling. Therefore, it was decided to use two consecutive extraction steps. The first extraction was performed with acetonitrile/water/formic acid (80:19.9:0.1, *v*/*v*/*v*) for 60 min, followed by a faster extraction for 30 min with acetonitrile/water/formic acid (20:79.9:0.1, *v*/*v*/*v*). A drawback of this procedure is that more matrix compounds are extracted because of the high water content of the second extraction solvent. However, the use of internal standards efficiently compensated all matrix effects for all target analytes.

On an economically important level, fumonisins are mainly produced by *Fusarium verticillioides*, a fungus predominant on maize and maize-based products [1]. In contrast to Lattanzio et al. [35], who concentrated on cereal products only, our aim was to develop a SIDA method capable of determining all mycotoxins regulated in maize and cereal-based foods; hence, special care regarding fumonisins was necessary. For method development, maize was chosen as the model matrix because most of the regulated mycotoxins are regulated in maize and the availability of accurate multi-mycotoxin methods for the analysis of maize is of great interest. Furthermore, none of the present multi-target methods using stable isotope dilution assays covers all mycotoxins regulated in maize. On top of that, maize is considered as a very complex matrix and is well known for its severe matrix effects compared with other cereals. It is very likely that a method performing well for maize is also applicable to other cereals and cereal-based foods, including baby food. Certainly, this has to be shown by additional validation studies. The only two mycotoxins which are regulated in the European Union [3–6] and not included in the method are aflatoxin M_1_ and patulin. Aflatoxin M_1_ is only regulated in milk and milk-based infant foods, and patulin is of interest in fruit juices and apple-based products, including apple compote, puree and apple-based baby food. For these liquid or high-water-content foods, a different sample preparation procedure is necessary.

### Evaluation of extraction recovery and matrix effects

For the evaluation of apparent recovery, extraction recovery and matrix effects, blank maize samples were spiked with working solution (unlabelled mycotoxins) on six levels in triplicate before as well as after extraction. The apparent recoveries of the whole method were determined using both internal and external calibrations (Table 2). In the case of external calibration, the obtained apparent recoveries for aflatoxins and DON ranged just between 35% and 50%, whereas enhanced values between 127% for T-2 and 356% for FB_1_ were observed. For ZEN, the only metabolite measured in the negative ionisation mode, no major alteration of the analytical signal was observed (*R*_A_, ext. = 89 ± 10%). Evaluation of the data with internal calibrations showed that the applied [^13^C] internal standards were capable of compensating all matrix effects efficiently (apparent recoveries of all 11 mycotoxins between 88% and 105%) and fulfil the requirements of EU legislation [36, 40]. The absence of matrix effects for the SIDA method was further checked by the evaluation of the spiked extracts after extraction with internal calibration (values of 90–98% were obtained). The determined relative standard deviations (RSDs) under repeatability conditions of the whole SIDA method were between 4% and 11% for all analytes, which is in compliance with Commission Regulation (EC) 401/2006 [40]. The extraction recovery was then calculated by dividing the apparent recoveries obtained from the samples spiked before extraction through those spiked after extraction (both with internal calibration). As shown in Table 2, the determined extraction recoveries were high for all mycotoxins (97–111%); even for FB_1_ and FB_2_, 103% and 95%, respectively, were achieved.

**Table 2:** Method performance parameters determined in maize

	LOD (μg kg^−1^)	LOQ (μg kg^−1^)	Eval. conc. range (μg kg^−1^)	*R*_A_ ext.^a^, *x* ± RSD	*R*_A_ int.^b^, *x* ± RSD	SSE int.^c^, *x* ± RSD	*R*_E_^d^, *x* ± RSD
AFB_1_	0.04	0.1	0.5–150	35 ± 5	105 ± 6	97 ± 4	108 ± 7
AFB_2_	0.04	0.1	0.5–150	45 ± 5	100 ± 4	93 ± 4	107 ± 6
AFG_1_	0.02	0.1	0.5–150	50 ± 4	101 ± 5	92 ± 4	109 ± 6
AFG_2_	0.1	0.4	0.5–150	43 ± 8	101 ± 8	91 ± 4	111 ± 9
DON	3.4	11	15–1,500	49 ± 6	96 ± 5	91 ± 4	106 ± 6
FB_1_	1.4	4.3	5–498	356 ± 10	101 ± 10	98 ± 9	103 ± 13
FB_2_	1.3	3.9	5–501	180 ± 8	88 ± 7	90 ± 7	97 ± 10
HT-2	0.8	2.5	4–1,340	148 ± 7	98 ± 7	90 ± 4	109 ± 7
OTA	0.1	0.4	0.5–153	168 ± 11	93 ± 7	91 ± 10	102 ± 12
T-2	0.1	0.2	0.5–151	127 ± 5	99 ± 6	90 ± 6	110 ± 8
ZEN	1.2	2.9	5–504	89 ± 10	103 ± 11	94 ± 12	109 ± 15

^a^Apparent recovery using external calibration

^b^Apparent recovery using internal calibration

^c^Mean value for the signal suppression or enhancement

^d^Extraction recovery

Several publications dealing with stable isotope dilution assays do not present data on matrix effects (e.g. [27]). When apparent recoveries of about 100% are reached, it is believed that the matrix effects are efficiently compensated. Nonetheless, severe matrix effects lead to a decrease in sensitivity as the LOQ cannot be improved by the use of internal standards. We expected severe matrix effects for the developed method because of a universal extraction (using high water content for the second extraction step), no sample cleanup, a minimal dilution of the raw extract and a comparable fast UHPLC-MS/MS method. The use of internal standards compensated matrix effects, influencing the apparent recovery, efficiently. Using a set of seven standards and three blanks (one before and two after the standard measurements), a sample throughput of over 90 samples per day is feasible with the presented method.

### LOD, LOQ, trueness

**Fig. 2:**
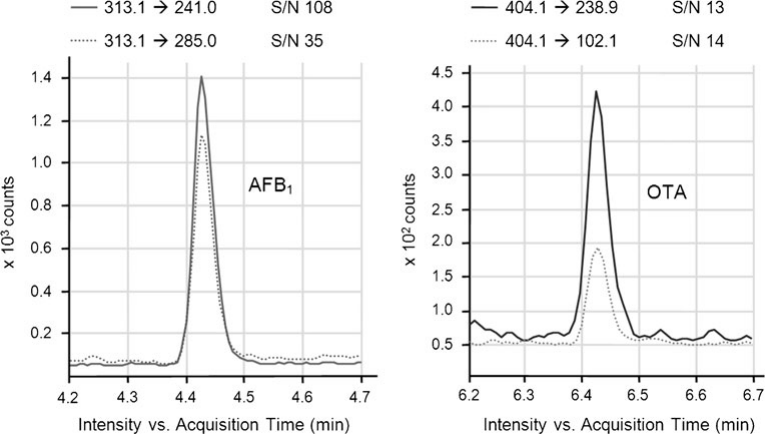
Extracted ion chromatogram of AFB_1_ and OTA of a blank maize sample spiked with 0.45 μg kg^−1^ before extraction and the corresponding signal-to-noise values for the two mass transitions

The LOD and LOQ of the method were derived by S/N ratios of 3:1 and 10:1, respectively, of spiked maize samples. The calculation was based on the less sensitive mass transition because the determination of a ratio between two mass transitions is required according to Commission Decision 2002/657/EC [36]. The obtained values shown in Table 2 were compared with the MLs specified in the European legislation [3–6], which has the strictest levels worldwide. The determined LOQs of the whole method in maize were far below the MLs for maize and cereal-based foodstuff. The method would even be applicable for the analysis of baby food assuming similar signal suppression and extraction recovery as maize, which is conceivable. The MLs for the critical mycotoxins, AFB_1_ and OTA, in baby food are close to the LOQ. Figure 2 shows the chromatogram as well as the calculated S/N ratios for a spiking level of 0.45 μg kg^−1^. For aflatoxins, the ML in processed cereal-based baby food and baby food for infants and young children as well as dietary foods for special medical purposes is 0.1 μg AFB_1_ per kilogram. The sum of aflatoxins (AFB_1_, AFB_2_, AFG_1_, AFG_2_) is not regulated in this category, and the lowest value is 4.0 μg kg^−1^ for cereals, with the exception of maize and rice prior to sorting (10.0 μg kg^−1^). In the case of OTA, the lowest value set in processed cereal-based food and baby food for infants and young children as well as dietary food for special medical purposes for infants is 0.5 μg kg^−1^. For processed (3.0 μg kg^−1^) and unprocessed (5.0 μg kg^−1^) cereal products, these values are higher and well above the LOQ of the presented method. The obtained sensitivity of the presented method is better than previously published methods [34, 35]. In contrast to [34], a triple quadrupole mass spectrometer was used instead of a high-resolution mass spectrometer, leading to higher sensitivity. The evaluated working range comprises a minimum of two orders of magnitude, which suits the analysis of mycotoxins in different foodstuff.

So far, no reference material with a certified concentration of all regulated mycotoxins is available. Therefore, 12 maize test materials with well-defined analyte concentrations covering 8 of the 11 target analytes were chosen and analysed to verify the trueness of the method. Table 3 summarizes the assigned values as well as the standard deviations according to the material provider. Furthermore, the values determined by the presented SIDA-UHPLC-MS/MS method as well as the standard deviation derived by the validation are given. In most cases, the measurement values fit the assigned ones within the respective uncertainties, proving the trueness of the method. In some cases, the determined results for fumonisins are higher than the specified range. This could be a result of the sample preparation and the use of an extraction solvent enabling high extraction recoveries of fumonisins. Although the results gained by inter-laboratory comparison studies should be corrected for recovery, it is expected that this might not have been sufficient in several cases [41]. For AFG_1_, twice the determined value is slightly higher than the assigned value of <0.1 μg kg^−1^; the AFB_1_ and AFB_2_ values are once lower than the assigned range.

**Table 3:** List of measured test materials together with assigned values and variation

No.	Analyte	Assigned value^a^ ± SD (μg kg^−1^)	Measured value^b^ ± SD (μg kg^−1^)	Status^c^
TM_01	ZEN	83 ± 4.5	86 ± 10	ok
TM_02	Sum AFs	3.79 ± 1.67	4.6 ± 0.2	ok
	AFB_1_	1.87 ± 0.83	2.3 ± 0.1	ok
	AFB_2_	0.51 ± 0.23	0.6 ± 0.03	ok
	AFG_1_	0.96 ± 0.43	1.0 ± 0.1	ok
	AFG_2_	0.52 ± 0.23	0.7 ± 0.1	ok
TM_03	FB_1_	1,650 ± 53	1,960 ± 198	+
	FB_2_	461 ± 16	496 ± 32	ok
TM_04	DON	1,714 ± 64	1,660 ± 145	ok
TM_05	DON	901 ± 55	908 ± 79	ok
	ZEN	79 ± 13	84 ± 10	ok
TM_06	FB_1_	2,630 ± 370	2,300 ± 233	ok
	FB_2_	690 ± 170	578 ± 38	ok
TM_07	FB_1_	270 ± 55	223 ± 23	ok
	FB_2_	<80	55 ± 4	ok
TM_08	Sum AFs	16.32 ± 2.03	13.3 ± 0.8	−
	AFB_1_	15.47 ± 1.97	12.5 ± 0.8	−
	AFB_2_	0.85 ± 0.17	0.6 ± 0.03	−
	AFG_1_	<0.1	0.2 ± 0.01	+
	AFG_2_	<0.1	<LOD	ok
TM_09	Sum AFs	8.9 ± 0.13^d^	8.3 ± 0.4	−
	AFB_1_	7.4 ± 0.19	7.4 ± 0.4	ok
	AFB_2_	0.7 ± 0.04	0.5 ± 0.2	ok
	AFG_1_	<0.1	0.4 ± 0.2	+
	AFG_2_	<0.1	<LOD	ok
TM_10	Sum FB_1_ + FB_2_	534 ± 33	598 ± 50	ok
	FB_1_	442 ± 27	490 ± 50	ok
	FB_2_	91 ± 6	108 ± 7	+
TM_11	Sum FB_1_ + FB_2_	1,194 ± 62	1,180 ± 99	ok
	FB_1_	987 ± 54	967 ± 98	ok
	FB_2_	197 ± 10	212 ± 14	ok
TM_12	Sum FB_1_ + FB_2_	1954 ± 126	2,140 ± 183	ok
	FB_1_	1,626 ± 108	1,800 ± 182	ok
	FB_2_	328 ± 22	343 ± 22	ok

^a^Assigned values and standard deviations (SDs) according to the material provider. If the standard deviation (or individual measurement results) was not given, the expanded uncertainty was divided by 2 to calculate the standard uncertainty

^b^Value measured by the developed SIDA-LC-MS/MS method ± SD calculated from the validation data

^c^The status indicates whether the determined range (measured value ± SD) is below (−), within (ok) or above (+) the specification range (assigned value ± SD)

^d^The sum of certified values for AFB_1_, AFB_2_, AFG_1_, AFG_2_ is not equal to the certified value of the sum of aflatoxins due to the different numbers of laboratories contributing to the determination

## Conclusion

LC-MS/MS multi-target methods are widely used for the determination of mycotoxins, but matrix effects may lead to inaccurate or even false results. The presented stable isotope dilution assay for aflatoxins (AFB_1_, AFB_2_, AFG_1_, AFG_2_), fumonisins (FB_1_, FB_2_), deoxynivalenol, ochratoxin A, zearalenone, HT-2 and T-2 toxin applies U-[^13^C]-labelled analogues of all target analytes prior to UHPLC-MS/MS analysis. This application minimizes the costs, compensates matrix effects efficiently and enhances overall accuracy. The required additional costs for the application of the [^13^C]-labelled internal standards in this application varied between EUR 0.02 for HT-2 and T-2 and EUR 0.48 for FB_2_ and resulted in total costs of below EUR 2 for all mycotoxins. This is quite low compared with immunoaffinity columns, which are not applicable for the determination of all investigated mycotoxins and still result in matrix effects. Sample preparation based on two extraction steps is fast, easy, and cheap and leads to very good extraction recoveries of 97–111% for all investigated mycotoxins. The “dilute-and-shoot” approach without any sample cleanup after extraction enables the simultaneous analysis of analytes with different chemical and physical properties. The application of fast polarity switching during UHPLC-MS/MS allowed the determination of the mycotoxins in the most abundant ionisation mode in one chromatographic run and reduced the run time to 11.5 min without losing sensitivity. The method fulfils the criteria set by the EU regulation concerning recovery and precision data [36] and performance criteria [40]. Furthermore, it is the first time that a multi-target method is capable of determining all mycotoxins regulated in the European Union for maize [3–6]. Due to the simple sample preparation, the fast analysis time and the compliance with EU regulation, this method is suitable for routine analysis.
